# Preservation of fertility following abnormally adherent placenta treated conservatively: a case report

**DOI:** 10.1186/1757-1626-2-9349

**Published:** 2009-12-18

**Authors:** Rajiv Mahendru, Bal K Taneja, Savita Malik

**Affiliations:** 1 Department of Obstetrics & Gynecology, MMIMSR, Mullana, Ambala, Haryana, 133203, India

## Abstract

**Introduction:**

One of the ensuing complications of placenta accreta includes loss of fertility.

**Case presentation:**

An Asian origin Indian national patient with history of placenta accreta at the time of previous delivery and had conservative management with injection methotrexate after the failure of surgical intervention, conceives again and has uneventful antenatal period and parturition.

**Conclusion:**

Conservative strategy of leaving the excessively adherent placenta in-situ alongwith adjuvant therapy in the form of injection methotrexate, not only prevents dreadful complications but also retains fertility in haemodynamically stable patients desirous of future pregnancy.

## Introduction

One of the potentially catastrophic obstetric complications, Placenta accreta is alarmingly on the rise, with high maternal morbidity and mortality rate being as high as 7% [1]. It is considered as an anomaly in placentation leading to its abnormally firm attachment to the myometrium due to the absence of decidua basalis leading to its incomplete separation at the time of delivery. The untoward complications may include- severe post-partum haemorrhage with its resultant coagulopathy, uterine perforation, shock, infection, loss of fertility and even death [2].

## Case presentation

A patient aged 33 years of Asian origin Indian national (with Hindu religion), reported missing her menstruation by 10 days and tested positive for pregnancy. Her obstetric history read third gravida with parity two, but one living child. The last time she was pregnant she had retained placenta following full-term vaginal delivery at home, complicated by post-partum haemorrhage.

Thereafter, being taken to a private hospital, she underwent three unsuccessful attempts of manual removal and uterine curettage and five units of blood transfusion interspersed with repeated episodes of excessive bleeding per vaginally. Failing to be managed by this surgical approach, she was referred for hysterectomy. Her first gestation period and vaginal delivery were uneventful.

On admission at Maharishi Markendeshwar Institute of Medical Sciences and Research (MMIMSR) after 18 days of parturition, she was conscious, cooperative, moderately built with body weight-50 kg, having stable general condition and normal vitals but with significant pallor, no cyanosis and nothing abnormal detected on respiratory or cardio-vascular system examination. On abdominal examination- uterus was felt enlarged to 20 weeks size, well contracted with Pelvic examination showing moderate amount of bleeding per vaginum and patulous but closed os of 20-22 wks sized uterus.

**Figure 1:**
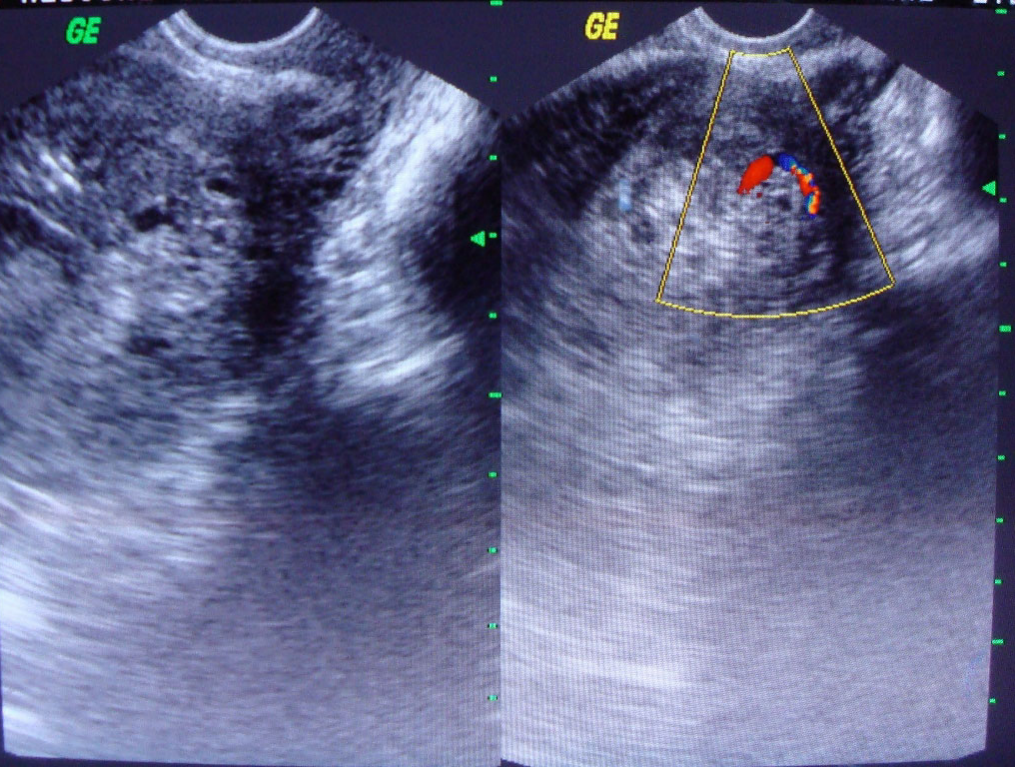
**Abnormally adherent placenta**.

Investigations revealed her Haemoglobin to be 7.5 gm%, blood group B positive; rest of the haemogram, urine routine examination, platelet count, coagulation profile, liver function and renal function tests were normal. Vaginal swab was sent for culture and sensitivity which later reported sterile. Sonography of abdomen revealed uterus to be enlarged with endometrial cavity showing an echogenic mass of dimensions 8.1 cm × 5.8 cm attached posteriorly, suggestive of placenta, with Colour Doppler confirming it to be posterior, low lying placenta accreta with no definite myometrial invasion (Figure 1).

Supportive measures, like one unit blood transfusion and broad-spectrum antibiotics, initiated. Considering the haemodynamically stable condition and minimal vaginal bleeding, she was managed conservatively. Modality adopted: placenta left insitu and injection methotrexate given intramuscularly in the dosage of 1 mg/kg body weight (i.e. single dose of 50 mg) repeated at 72-96 hourly intervals for a total three doses depending upon the dimension and vascularity of the endometrial mass representing adherent placenta with serial Ultrasonography and colour Doppler studies which showed gradual reduction. Total and Differential Leucocyte counts were routinely done and remained within normal limits. Size of the uterus decreased remarkably and was not palpable per-abdomen after 7 days. Vaginally, bleeding never became alarming and the discharge never appeared infected.

Patient was discharged satisfactorily after 11 days. On subsequent three follow-up visits, every 5 days, patient remained afebrile with no evidence of infection, and near normal ultrasonographic findings after a fornight. Patient started menstruating regularly after about four months. Present pregnancy happened after 8 months of the above mentioned previous eventful post-natal period. In her regular antenatal visits, Ultrasonography and colour Doppler were conducted at frequent intervals to rule out any evidence of recurrent Placenta accreta. At term, she had caesarean section for foetal indication. The placental separation was normal and post natal period uneventful

## Discussion

The ever increasing incidence of life endangering condition of placenta accreta is considered between 1 in 7000 to as high as 1 in 540 pregnancies [3]. The risk factors for placenta accreta are -previous uterine surgery(like caesarean sections, Myomectomy), previous dilation & evacuation, placenta praevia, advanced maternal age, multiparity, Asherman's Syndrome and presence of submucous leiomyomata [4,5]. It is important to make an early and accurate diagnosis by Ultrasound, Colour Doppler, MRI for appropriate management and reduction of associated morbidity, thereof [6].

Two strategies for the management of placenta accreta have been described: surgical removal of the uterus and conservative management. Presently, there has been a gradual shift towards its conservative management pioneered by Arulkumaran et al [7], away from the age old traditional approach of hysterectomy. The current trend is of uterine conservation and leaving the adherent placenta in-situ with adjuvant treatment with Methotrexate [8]. Mussali et al [9], in 2000, managed three cases of placenta accreta with methotrexate and succeded in preserving the uterus in two cases. One case of placenta percreta and three cases of partial placenta increta were managed effectively with methotrexate by Sonin in 2001 [10], and Pinho et al in 2008 [11], respectively.

Another study on conservative management mentions leaving the placenta accreta in situ with one of these associated treatments like -bilateral hypogastric artery ligation, medical treatment with Methotrexate or uterine artery embolisation,: placental resorption happened in majority of the cases with no report of maternal mortality but two cases failed where hysterectomy was performed -in one case for life threatening haemorrhage and in the other for post-embolisation uterine necrosis [12].

Though uterine artery embolisation may be regarded as an alternative to surgery for control of obstetric haemorrhage in placenta accreta [13], its safety for women desiring future pregnancy is controversial [14].

According to Heiskanen et al [15], conservative management of adherent placenta with methotrexate to preserve future fertility remains a secure and reasonable alternative in haemodynamically stable patients when there is no active bleeding.

## Conclusion

Conservative strategy of leaving adherent placenta in-situ with adjuvant methotrexate deserves consideration in selected patients of placenta accreta who are haemodynamically stable and with no active bleeding, especially in those category where retention of fertility is required.

## Consent

Written informed consent was obtained from the patient for publication of this case report and accompanying images. A copy of the written consent is available for review by the Editor-in-Chief of this journal.

## Competing interests

The authors declare that they have no competing interests.

## Authors' contributions

RM managed the patient and a major contributor in writing the manuscript. BKT provided the necessary supervision. SM helped in analyzing the patient data. All authors have read and approved the final manuscript.
